# Digital competency of Psychologists in Aotearoa New Zealand: A cross-sectional survey

**DOI:** 10.3389/fdgth.2022.951366

**Published:** 2022-09-07

**Authors:** Rosie Dobson, Rushaina Variava, Meihana Douglas, Lisa M. Reynolds

**Affiliations:** ^1^ National Institute for Health Innovation, University of Auckland, Auckland, New Zealand; ^2^ Institute for Innovation and Improvement, Waitematā District Health Board, Auckland, New Zealand; ^3^ Department of Psychological Medicine, University of Auckland, Auckland, New Zealand; ^4^ National Hauora Coalition, Auckland, New Zealand

**Keywords:** digital health, psychology, skills & competencies, professional standards for psychological service

## Abstract

**Background:**

The increasing implementation of digital health into psychological practice is transforming mental health services. Limited clinical resources and the high demand for psychological services, alongside the restrictions imposed on services during the global COVID-19 pandemic, have been a catalyst for significant changes in the way psychologists work. Ensuring Psychologists have the skills and competence to use these tools in practice is essential to safe and ethical practice.

**Aim:**

This study aimed to explore the digital competence of psychologists working in Aotearoa New Zealand and their use of digital tools in the practice.

**Methods:**

A cross-sectional online survey was conducted with Aotearoa New Zealand Registered Psychologists (*n* = 195) between July and November 2021.

**Results:**

Participants reported varying degrees of competence across the digital tasks presented, with participants most commonly reporting moderate to high competence for engaging in remote supervision *via* digital means (86%) and obtaining client's informed consent for digital work (82%). In contrast, tasks that participants most reported not being moderately or highly competent in included working with interpreters remotely and evaluating the effectiveness and security of smartphone apps. Motivations to use digital technologies included meeting client preferences and needs, necessity for continuity of care, and the benefits of increased accessibility and reach. In contrast, the barriers to using digital technologies included client characteristics or preference, clinical factors, clinician preferences and skills, and workplace or technical issues or concerns. The majority (91.1%) were potentially interested in further training in this area.

**Conclusions:**

The current study offers insights into the digital competencies of a workforce that has required rapid incorporation of technologies into professional practice over recent years. This snapshot of the digital skills of psychologists demonstrates a large variation in digital competence. In the current context, developing digital competencies seems a fundamental requirement for psychologists to work in ways that appropriately and safely deliver client-centred care.

## Introduction

The context of psychological practice is swiftly transforming. Rapid advances in digital health, limited clinical resources and the high demand for psychological interventions are changing the face of psychological practice (1, 2). Technologies identified as having the potential to impact mental healthcare range from ones now common place such as telehealth and social media, through to artificial intelligence and virtual or augmented reality (3). These developments in technology present opportunities to connect and empower clients as well as create strategies to meet the increasing demand for psychological services (3–5). Further digital health has the potential to transform processes around assessment, diagnosis, and monitoring by providing supplementary insights into behaviours and activities (4). Although the potential benefits are vast, previously identified barriers to the uptake and use of digital technologies in clinical practice include workplace/environmental factors (e.g., availability of technology and support to use it), clinician factors (e.g., digital competency, willingness to use technology), technology factors (e.g., ease of use, perceived benefits), and client characteristics (e.g., access to technology and ability to use it) (6–9).

Digital health has been rapidly implemented into modern-day psychological practice, with the COVID-19 pandemic acting as a catalyst and influencing the magnitude of change (10, 11). The COVID-19 pandemic and its associated lockdowns resulted in a sudden transition from in-person practice to online practice for many (10). The rapid developments and consistent use of these technologies in our everyday lives, further strengthened their diffusion into healthcare provision and the shift from traditional, in-person modalities to digital modalities (2, 12–14). Within this rapidly changing landscape of psychological practice, it is critical for psychologists to hold adequate digital competencies to conduct psychological practice safely and ethically (2, 14). Although the changes made to clinical practice during the global pandemic may not be permanent it has highlighted that psychologists must be adaptable and flexible within their practice to ensure that they can uptake these technologies when needed and maintain their competencies (1). Psychologists must exercise their clinical judgement to respond with the best outcomes for their clients in mind (15). There are no current formal competencies related to digital practice for New Zealand registered psychologists (16), and little is known about the current workforces competence in this area.

This study was designed to explore the digital competence of psychologists working in Aotearoa New Zealand and their use of digital tools in the practice. The overarching aim of this study was to acquire an understanding of the digital health competence currently held by the psychologist workforce. A secondary aim was to explore the enablers and barriers to use of digital tools in clinical practice.

## Materials and methods

### Study design

A descriptive cross-sectional survey was conducted with New Zealand Registered Psychologists. The description of the survey is described according to CHERRIES (17). Ethical approval for this study was obtained from the Auckland Health Research Ethics Committee (AHREC) on 09/03/2021 for three years (REF: #AH22139).

### Survey design

The study involved an anonymous questionnaire which incorporated closed and open-ended questions designed to capture information about digital competence and utilisation of digital technologies. The survey included:

‐ Screening questions to confirm eligibility (e.g., registration status)
‐ Demographics and questions about professional psychological practice (e.g., number of years practicing as a psychologist)
‐ Rating of competency on tasks related to practicing psychology digitally
‐ Factors which influence use of digital technology in practice
‐ Motivators and barriers to using digital technologies in practice
‐ Interest in further training on use of digital technologies

The survey also included measures of compassion and burnout which are not reported in this paper.

The survey was designed in paper format and uploaded into an electronic format for administration. The average time to complete the questionnaire was 15 min. The survey was pre-tested by researchers and members of the target population before being finalised.

The survey was identical for all participants (no randomized items), and participants were able to go back and change their responses before submission.

### Inclusion criteria

The inclusion criteria for the study were that participants need to (1) be New Zealand registered psychologists (under any scope of practice, including interns), (2) hold an annual practicing certificate, (3) work in Aotearoa New Zealand and (4) speak English. Psychologists who were not currently registered and/or did not hold an annual practicing certificate were excluded from the study.

### Procedures

All psychologists who fit the inclusion criteria were eligible to participate. Convenience sampling methods such as professional mailing lists, professional social media groups and word of mouth were used to recruit prospective participants. Online study advertising and emails about the study contained the link for the questionnaire which was hosted by the online platform Qualtrics. When participants clicked on the link they were redirected to information about the study and details of who to contact if they had questions and were asked to confirm their consent to participate before they were able to access/complete the survey. Participation was entirely voluntary, and at the conclusion of the survey participants could opt to be entered into the draw to win an iPad.

### Measures

At the time of this study there were no mandated digital competency requirements for New Zealand Psychologists (16). and there was no validated measure of digital competence or digital health literacy relevant to psychological practice available. Therefore a measure was developed specifically for this study based on the digital competencies developed by the British Psychological Society (18). A group of clinical and academic psychologists with a range of experience in training psychologists on core competencies, digital skills, developing psychometric measures, and working with the indigenous population of New Zealand (Māori), reviewed the British Psychological Society competencies to generate a list of tasks relevant to explore the digital health competence of psychologists working in Aotearoa New Zealand. To ensure relevance to psychologists working in Aotearoa, where competency in working with Māori is a requirement (16), culturally relevant items and Te Reo Māori terms (e.g., “whānau”) were included in the list. Initial items were developed and piloted before being further refined by the expert group and the final 41-item Digital Competency Scale (DCS) confirmed. Psychologists were asked to rate their ability to conduct each of the tasks on a scale from 1 to 5 (1 = not competent; 2 = slightly competent; 3 = somewhat competent; 4 = moderately competent; 5 = very competent).

To assess enablers and barriers of digital technology uptake, participants were asked to rate how client characteristics, clinical factors, workplace requirements, technology factors and personal preferences influence their use of digital technologies on a scale of 1 (no influence) to 5 (major influence). Participants were also asked two open-ended questions about (1) the motivators and (2) the barriers to using digital technologies in psychological practice. Finally, participants were asked if they would like further training on using digital technologies (yes/no/maybe).

### Statistical analysis

Analyses were conducted with the Statistical Package for the Social Sciences (IBM SPSS Statistics V.26) software. Survey data were analysed and summarized using descriptive quantitative analyses. To provide clarity in interpretation, items on the DCS were collapsed into three categories; 1 (not/slightly), 2 (somewhat), 3 (moderately/very) with numbers and percentages of responses calculated for each item. The means and standard deviations of items relating to enablers and barriers of using digital tools in clinical practice were calculated. Qualitative comments were analysed using a thematic analysis (19). This approach identifies common themes and meanings from the data. A description of the themes is provided and quotes presented as examples of the themes. Quantification of the themes was not undertaken. Ethnicity was coded as per New Zealand Ministry of Health Protocol for the reporting of ethnicity data, with the “prioritised ethnicity” output method used for reporting in this paper (20).

No timeframe was imposed on participants to complete the questionnaire, cookies were not used to assign identifiers to each computer, and IP address information was not recorded. Completeness checks of responses were completed after submission. For a questionnaire to be considered complete participants needed to have completed to the end of the Digital Competency Scale. Digital Competency Scale items were mandatory, but all other questions were optional. Adaptive questioning was not used within the questionnaire. View rate of the questionnaire was not recorded. Multiple submissions were prevented on Qualtrics and further manual checks for multiple entries were also performed.

## Results

There was a total of 252 people visit the survey and provide consent to participate. Of the 248 who completed screening 6 were not eligible for the study (were not currently registered). A total of 195 psychologists completed the survey between 20 July and 12 November 2021. The sample is representative of approximately 6% of the total psychologist workforce (*N* = 3,199) in Aotearoa with a current practicing certificate (21). The completion rate of the questionnaire was 77%.

### Demographic and professional information

The demographic and professional breakdown of respondents can be seen in Table 1. The sample were predominantly New Zealand European (*n* = 129; 66%) and female (*n* = 171; 88%), and the majority were registered under the general psychologist (40%) and clinical psychologist (39%) scopes of practice. Although nearly half of the sample had only been practicing for up to 5 years, one fifth had been practicing for over 20 years.

**Table 1:** Demographic and professional characteristics of respondents (*n* = 195).

Characteristic	*N*	%
Ethnicity		
European	154	79%
Māori	17	9%
Asian	16	8%
Middle Eastern, Latin American, African	4	2%
Other Ethnicity	4	2%
Age group		
24–35	72	37%
36–45	59	30%
46–55	33	17%
56–65	20	10%
Over 65 years	11	6%
Gender		
Male	24	12%
Female	171	88%
Scopes of practice		
Psychologist	78	40%
Clinical psychologist	75	39%
Intern psychologist	25	13%
Other	17	8.7%
Primary work setting		
District health board (DHB)	59	29%
Primary health organisation (PHO)	8	4%
Non-government organisation (NGO)	13	7%
Private practice	64	33%
Forensic setting (e.g., prison)	17	9%
Other (e.g., Education, Government)	34	17%
Years of practice		
≤5	84	43%
6–10	29	15%
11–20	44	23%
21–29	19	10%
≥30	19	10%

### Digital competency

The number of participants reporting little or no competence, some competence, or moderate to very competent across all the digital competence items is presented in Table 2. Over three quarters of participants reported moderate to high competence in engaging in remote supervision *via* digital means (86%), obtaining client's informed consent for digital work (82%), managing professional and clinical boundaries related to online practice (77%), establishing and maintaining a positive therapeutic relationship online and with telephone work (76%), following organisational policies and procedures related to digital work (76%), and reflecting on one's own attitudes, skills and values regarding digital practice (76%).

**Table 2:** Rating of level of competence on digital tasks (*n* = 195).

Tasks	None to slightly competent (1 or 2)	Somewhat competent (3)	Moderate to very competent (4 or 5)
	*n* (%)	*n* (%)	*n* (%)
Establish and maintain a positive therapeutic relationship online and with telephone work	11 (6%)	36 (19%)	148 (76%)
Consider the advantages and drawbacks of digital tools with reference to the evidence base	40 (21%)	56 (29%)	99 (51%)
Reflect on your own digital psychological practice	17 (9%)	49 (25%)	129 (66%)
Recognise your competencies, training and supervision needs in relation to digital practice	21 (11%)	55 (28%)	119 (61%)
Provide culturally appropriate materials and interventions using digital resources	85 (44%)	60 (31%)	50 (26%)
Manage professional and clinical boundaries related to online practice	12 (6%)	33 (17%)	150 (77%)
Obtain the client's informed consent for digital work	13 (7%)	23 (12%)	159 (82%)
Follow organisational policies and procedures related to digital work	15 (8%)	32 (16%)	148 (76%)
Select online psychological assessments that are suitable for remote administration	64 (33%)	53 (27%)	78 (40%)
Administer online psychological assessment tools *via* remote means	76 (39%)	51 (26%)	68 (35%)
Conduct accurate risk and clinical safety assessments using digital technologies	47 (24%)	49 (25%)	99 (51%)
Assess and match client needs, interests, and abilities to suitable digital modalities	44 (23%)	64 (33%)	87 (45%)
Assess a client's suitability for online interventions	31 (16%)	55 (28%)	109 (56%)
Work ethically and safely in digital practice	18 (9%)	39 (20%)	138 (71%)
Recommend appropriate online resources to my clients	15 (8%)	54 (28%)	126 (65%)
Use a wide range of digital technologies to help my learning (e.g., e-learning modules)	26 (13%)	42 (22%)	127 (65%)
Work collaboratively with a client remotely e.g. using screen sharing	39 (20%)	39 (20%)	117 (60%)
Conduct individual therapy using digital technologies	25 (13%)	42 (22%)	128 (66%)
Conduct group therapy using digital technologies	120 (62%)	30 (15%)	45 (23%)
Adapt digital interventions to the needs of clients	48 (25%)	58 (30%)	89 (46%)
Involve whānau in online and telephone work	66 (34%)	59 (30%)	70 (36%)
Recognise how digital technologies may influence confidentiality and its limits e.g. security of recordings	16 (8%)	42 (22%)	137 (70%)
Manage data collected digitally and integrate this into treatment planning	44 (23%)	61 (31%)	90 (46%)
Evaluate the effectiveness and security of smartphone apps	95 (49%)	58 (30%)	42 (22%)
Reflect in supervision on the client's response to different digital modalities	32 (16%)	47 (24%)	116 (60%)
Introduce and support the use of self-help digital tools for clients to use	40 (21%)	65 (33%)	90 (46%)
Integrate visual digital tools to complement online interventions e.g. using shared documents	67 (34%)	56 (29%)	72 (37%)
Adapt evidence-based interventions to online delivery	46 (24%)	58 (30%)	91 (47%)
Critically appraise digital resources for selection for use in clinical and research work	53 (27%)	80 (41%)	62 (32%)
Monitor client experience and client-reported outcomes using digital methods	55 (28%)	56 (29%)	84 (43%)
Discuss the pros and cons of the digital modality with the client	21 (11%)	61 (31%)	113 (58%)
Adapt your communication style depending on the technology used to promote the therapeutic relationship	20 (10%)	40 (21%)	135 (69%)
Work with interpreters remotely e.g. having an interpreter join a call to translate for a client	141 (72%)	28 (14%)	26 (13%)
Manage boundaries if working remotely e.g. conducting a therapy session *via* video conference from home	14 (7%)	39 (20%)	142 (73%)
Deliver e-learning through digital methods (e.g., eBooks, vlogs, live webinars)	91 (47%)	38 (20%)	66 (34%)
Engage in remote supervision *via* digital means	9 (5%)	18 (9%)	168 (86%)
Follow organisational policies and procedures in the making, storing, and sharing of recordings of sensitive clinical material	20 (10%)	37 (19%)	138 (71%)
Engage in leadership and consultation to promote digital practice amongst others	89 (46%)	51 (26%)	55 (28%)
Work in remote digital teams and participate in remote digital meetings	20 (10%)	37 (19%)	138 (71%)
Reflect on one's own attitudes, skills, and values regarding digital practice	11 (6%)	35 (18%)	149 (76%)
Recognise and reflect on the limits of one's own competence when translating in-person training to online work	24 (12%)	49 (25%)	122 (63%)

In contrast, less than one quarter of participants reported moderate to high competence in working with interpreters remotely (13%), evaluating the effectiveness and security of smartphone apps (22%), and conducting group therapy using digital technologies (23%).

There were 5 (3%) participants who reported that they were not competent (rating 1) on over half of the items. Conversely there were 21 (11%) participants who were very competent (rating 5) on over half of the items. Only 1 participant (0.5%) reported being at least somewhat competent across all tasks (ratings of 3, 4 or 5).

### Factors influencing the use of digital technologies

Participants reported that client characteristics such as client access to, and confidence with, technology was the biggest influence on their use of digital technologies in practice (see Table 3).

**Table 3:** Mean ratings of the degree factors influence the use of digital technologies in psychological practice (1 = No influence to 5 = Major influence; *n* = 191).

Factors	Mean (SD)
Client's characteristics (e.g., client's access to technology, client confidence with technology, client preference)	4.59 (0.79)
Clinical psychopathology (e.g., client diagnosis)	3.60 (1.21)
Workplace factors (e.g., access to digital tools in the workplace, workplace guidelines, workplace support)	3.81 (1.21)
Technology factors (e.g., security concerns, costs, technical support)	3.60 (1.07)
Personal factors (e.g., individual preferences, personal comfort with technology)	3.64 (1.13)

#### Motivations to use digital technologies

There were three overarching themes identified in regard to what motivates the use of digital technologies in psychological practice. These included (1) meeting client preferences and needs, (2) necessity for continuity of care, and (3) the benefits of increased accessibility and reach.

1. Client preferences and needs encompassed factors such as client convenience and flexibility, and their preferences to use digital technologies. Participants commented that they were motivated to use digital technologies to provide psychological services when clients were unwell or had mobility issues.
  *“If patients are unable to attend clinic (e.g., barriers such as work, transport, illness, COVID lockdowns) then it is a good way to still provide therapy and gives the patient more flexibility. With COVID lockdowns, some patients became quite anxious about coming into clinic and we were able to use digital technology to get around this.” [#29; Psychologist]*
  *“Client requests, distance, ease of meeting in person, health (I offer Zoom sessions if I or the client have cold/flu symptoms)” [#78; Clinical Psychologist]*
  *“Some of my clients live remotely. Some prefer not to commute to my office. Since Covid some choose to interact digitally if they or I have sick children at home from school. Continuing to offer support throughout lockdowns is also a motivation.” [#94; Psychologist]*
2. The necessity for continuity of care was also a key motivator for participants to utilise digital technologies in their practice. This was commonly related to the COVID-19 pandemic and the necessity to continue the provision of psychological services during the pandemic and associated restrictions (e.g., lockdowns) when they were unable to see clients in the face-to-face setting.
  *“It is a necessary medium for current psychological practice and it can remove barriers to access.” [#02; Psychologist]*
  *“The covid lockdown in 2020 prompted me to offer virtual sessions and I have incorporated this as an offering going forward. I am now able to continue therapy with clients if they move cities.” [#49; Psychologist]*
  *“COVID has really changed my way of working. In addition, I travel for work to see clients and so it is wise in terms of resources for me to alternate travel with digital technology use.” [#89; Educational Psychologist]*
3. The benefit of increased accessibility and reach of digital technologies was also a motivator for psychologists to use digital technologies in practice. Digital technology allowed them to overcome barriers of access to services arising from cost, time, and location. Providing equitable access to psychological services across Aotearoa New Zealand, including rural areas was motivating for many of the respondents.
  *“Increasing access to people who can*’*t access conventional services.” [04; Psychologist]*
  *“Increased accessibility for clients/reduces barriers, helps service increase offerings to clients in community, can do webinars/e-groups targeting larger proportion of community at once (vs. 1:1 therapy)” [#22; Clinical Psychologist]*
  *“Covering a wide geographical area it makes sense to complete some of my clinical work remotely to reduce travel. I also find it helpful to link in with colleagues around our (large) DHB and also across the South Island and nationally” [#34; Psychologist]*

#### Barriers to using digital technologies

Participants were also asked about the barriers to using digital technologies in practice. The key themes identified from the responses included (1) client characteristics or preference, (2) clinical factors, (3) clinician preferences and skills, and (4) workplace and technical issues or concerns.

1. Client characteristics were a common barrier to the use of digital tools including client preference and ability to use digital tools. Access to digital tools, particularly for those from low socioeconomic areas was also a barrier.
  *“Older patients do not always have the technology or skills to use the technology. Some patients do not have access to technology/wifi and are not financially able to use these technologies. If patients are at work or home, sometimes they are unable to find a private space to do a session. Sometimes getting the patient to come into clinic can be an intervention in itself and using digital technologies can become a barrier as patients may use this as an avoidance strategy.” [#29; Psychologist]*
  *“Client access to broadband. Client access to platform for online therapy. Client reluctance to do therapy online.” [#130; Clinical Psychologist]*
  *“Working with older clients (65+) often do not have the digital literacy to use technology appropriately” [#102; Clinical Psychologist]*
2. Participants also described clinical or cultural safety factors as barriers to using digital tools. These included concerns for clients at risk, concerns around client privacy, that digital tools were often unsuitable for certain patients (i.e., those who have intellectual disabilities) or types of therapies, and challenges engaging in cultural practices in the digital space.
  *“Accessibility, difficulty transferring the cultural practices into the digital marae e.g. through zui.” [#109; Intern Psychologist]*
  *“Not suitable for patients at risk, patients with limited privacy - unable to attend digital sessions if there is not a space for them to utilise i.e. have had patients go out to their car to gain privacy” [#31; Psychologist]*
  *“Clinical risk factors would stop me from doing telehealth consultations. Also the type of input required for a client. For example, I don*’*t do reprocessing sessions (EMDR) online and I prefer not to work with traumatised clients via online only sessions.” [#124; Clinical Psychologist]*
3. Some participants described personal factors as barriers to using digital technology in practice including not having the skills, confidence, or resources to utilise digital tools, and for some the lack of time to upskill in this area. Others described a preference for non-digital approaches and not wanting to upskill in this space.
  *“No barriers other than my preference to see face to face for a better interpretation of client's body language.” [#28; Clinical Psychologist]*
  *“My own knowledge and lack of time and interest in learning more.” [#96; Clinical Psychologist]*
  *“Personal dislike of excessive screen use and personally find it harder to connect with people online and harder to read the situation/client responses” [#187; Psychologist]*
4. Lastly, participants described a number or organisational or service-related barriers as well as technical issues preventing their use of digital tools. This included limited access to technology and adequate space to use it, lack of technology support, lack of training and security issues. Issues related to connectivity issues/speed and reliability concerns of digital tools were also mentioned.
  *“Security of DHB network, risk acuity of clients, privacy concerns.” [#82; Clinical Psychologist]*
  *“The DHBs current set—up (space issues, technology issues)” [#26; Psychologist]*
  *“IS support and security” [#72; Clinical Psychologist]*

#### Further training on the use of digital technologies

Of the 190 participants who completed the question about further training, 101 (53.2%) reported that they would like further training and a further 72 (37.9%) answered maybe. Only 17 participants (8.9%) reported they would not be interested in further training.

## Discussion

The current work offers insights into the digital competencies of a workforce that has required rapid incorporation of technologies into professional practice over recent years. This snapshot study into the digital skills of psychologists demonstrates a large variation in digital competence. Although the majority of participants reported at least moderate competencies in areas essential to effective and safe psychological practice, including their abilities to form a therapeutic alliance online, conduct individual therapy using digital technologies, reflect on their abilities, and manage ethical/professional responsibilities using digital technologies, there were important areas where competencies were notably lacking. For instance, people reported low digital competence in doing group therapy, evaluating apps, providing culturally appropriate interventions, and working with interpreters. Providing culturally safe intervention is of particular note and training psychologists in such areas seems a priority area for future focus. Our work has also identified key motivators and barriers to using digital technologies by psychologists. Similar to previous studies, clinician, client and practice factors impact psychologists use of digital tools (6–8). Of note, client preferences were a major influence on the decision to use such tools in our study and reflect an appropriate emphasis on client centered care. Many participants in our sample were aware that clients are often disadvantaged in accessing conventional “bricks and mortar” type services through difficulties with transport or geographical limitations and preferred to use technology for these reasons. Online delivery of psychological services can help to minimise the access barriers, particularly for those living rurally or with disabilities. Further, there is increasing evidence to support the use of digital tools to reduce healthcare inequities such as those experienced by Māori (NZ indigenous population) (22). It is worth noting, that such delivery can present different barriers to clients who do not have access to required technologies or have poor digital literacy skills. However, in the context of widening health inequities, ensuring psychologists are digitally competent has important implications in enabling care to be delivered through the medium that best meets client needs.

In line with previous surveys, the majority of participants in this study were interested in further training in digital health (6, 23). But this study has shown that a key barrier to the use of digital tools lies with psychologists themselves. Although most of our sample stated a willingness to upskill in this area, there were others who said they did not want to use digital tools nor upskill in this space. With clear equity implications in accessing psychology services compounded by the restrictions imposed on in-person care during the pandemic (and the associated risk to client safety of meeting in person), resistance to upskilling raises questions about whether some psychologists can safely deliver care in a way that best meets clients' interests. Given there is a section of the workforce unmotivated to develop new skills, one way to address such preferences and deficits in skills is for professional bodies to mandate the development of digital competencies as a requirement of competency more broadly.

### Limitations

Although this study provides important insights into the digital skills of psychologists in a fast-changing context, this work is not without limitations. First, our study design precludes our ability to comment on whether the areas where people lacked competency were particular to digital modalities or whether people might lack competency across other formats as well. For instance, we suspect that some of our findings reflected a more general lack of skills that is not confined to online work (e.g., difficulties in working with interpreters). Another issue worth noting is that we did not assess how *important* participants rated the various competencies. For example, some psychologists might lack skills in facilitating groups simply because their roles do not require it. Further we did not assess the level of training the participants had previously undertaken in this area which may have impacted their competency as well as willingness to undertake further training in this area. Another limitation of the current work is that our sample is small and may not be more widely representative of the psychologist population impacting the generalisability of the results. Although we utilised a wide variety of recruitment methods, our sample was only approximately 6% of the psychologist population in Aotearoa New Zealand and it is possible (if not likely) that psychologists who did not complete our online survey had poorer digital competency than their counterparts who engaged with the digital research. We also only offered the survey in English and psychologists who speak other languages may have had a different perspective, particularly in regard to cultural work. Further, we are unable to comment on the generalizability of our qualitative themes regarding motivators and barriers to digital technology uptake. Although quantification of qualitative data is possible this is not the approach we took. We believe that the broad, open-ended, format of the questions related to barriers and motivators means that counting responses would imply generalizability that is beyond the scope of our data. Future studies should consider quantifying the extent to which these themes might be apparent in the psychology workforce. Finally, it is important to note the timing of this survey which ran during a timeline of various geographical COVID-19 associated lockdowns. It is difficult to know what impact this might have had on responses, but one possibility is that psychologists may have been frustrated at this time by ongoing uncertainty in delivering their psychological services.

### Recommendations

In presenting the findings of this study, it is apparent that the development of digital competencies in psychologists is important for several reasons. Digital skills have a role in future-proofing the profession as technology advances, meeting the needs of clients through maintaining access and tailoring delivery to preferences to work with technology, and as a safeguard to future lockdowns or public health threats. We believe that registering bodies, psychological services, education providers, and psychologists themselves, all have a role to play in the development of these skills and outline several recommendations below.

First, we recommend that the regulatory bodies that provide assurance of competency in psychologists consider digital skills as a key tenant of safe and competent professional practice. We acknowledge that this has been a fast-moving space, however, such registering bodies have a duty to the public to ensure that psychologists are delivering competent care. At the time of writing, although the New Zealand Psychologist Board provides 2012 guidelines on “The Practice of Telepsychology” (currently “under review”) (24), there is no mandate for psychologists to maintain even a basic level of digital skills. It is important to note that other regulatory bodies have moved forward in this area, for example, the Occupational Therapy Board of New Zealand Competencies for Registration and Continuing Practice now include competencies specific to digital practice (25).

Second, we recommend that organisations providing psychological services (e.g., health services) review the way they support their workforce in their digital practice. Clearly, psychologists need access to adequate digital tools and technologies, a suitable (and confidential) space to use them, appropriate security measures and assurances, technological support, and the time and resources to develop these new skills. Arguably, organisations have an obligation to provide these resources and support to their workforce. Considering many of these organisations provide a range of clinical services, it is important that these considerations are not limited to the delivery of psychology services alone and that the ability to provide client/patient informed multidisciplinary care digitally in a safe and ethical way is prioritised. Furthermore, organisations have the opportunity to provide accessible targeted training to their workforce to ensure clinicians have the skills, knowledge and confidence to implement clinical IT change essential in the current context. An example of where this has been done successfully in Aotearoa New Zealand is the Waitematā District Health Board Clinical Digital Academy where clinicians receive training in a range of digital and clinical IT areas, including health information systems, artificial intelligence, ethics, data analytics, cybersecurity, and social media (26).

Third, we recommend that the education providers training students to become psychologists ensure that their curricula include training in the various digital competencies we outline in this work. The Topol report in 2019 recommended that digital literacy should be built into clinical training programmes to ensure development of specific competencies in the use of technology (9). Guidelines around digital practice such as those developed for NZ nurses entering clinical practice could help to ensure future psychologists enter the workforce competent in this area (27). Furthermore, this study has shown interest in training in digital skills from currently practicing psychologists and education providers are well placed to offer adjunct training for existing psychologists seeking to upskill.

Finally, we encourage psychologists themselves to reflect on their own digital competencies and to consider whether their current skill set meets the needs of their clients in this fast-changing context.

## Conclusions

The aim of the current work was to gain an understanding of the digital health competencies currently held by the psychologist workforce. In doing this, we have created a benchmark on which to compare future developments. Overall, although our sample was small, we found that psychologists rated themselves as moderately to very competent in many areas. However, in other areas, such as providing culturally appropriate interventions, competencies were lacking and at least some of our sample were unwilling to upskill. In the current context, developing digital competencies seems a fundamental requirement for psychologists to work in ways that appropriately deliver client-centred care. In closing, we note that although our focus was on one particular workforce, we suspect that these findings would translate to others and we call to action other healthcare providers to consider how digital competencies might be relevant in their contexts.

## Data Availability

The raw data supporting the conclusions of this article will be made available by the authors, without undue reservation, where appropriate approvals have been obtained.

## References

[1] Bucci S Schwannauer M Berry N. The digital revolution and its impact on mental health care. Psychology and psychotherapy: theory. Psychol Psychother. (2019) 92(2):277–97. 10.1111/papt.12222 30924316

[2] Morris ME Aguilera A. Mobile, social, and wearable computing and the evolution of psychological practice. Prof Psychol Res Pr. (2012) 43(6):622. 10.1037/a0029041 25587207 PMC4289629

[3] Foley T Woollard J. The digital future of mental healthcare and its workforce: A report on a mental health stakeholder engagement to inform the Topol Review. NHS Health Education England (2019). Available from: https://topol.hee.nhs.uk/wp-content/uploads/HEE-Topol-Review-Mental-health-paper.pdf (Accessed May 18, 2022).

[4] Hollis C Morriss R Martin J Amani S Cotton R Denis M Technological innovations in mental healthcare: harnessing the digital revolution. Br J Psychiatry. (2015) 206(4):263–5. 10.1192/bjp.bp.113.142612 25833865

[5] Kazdin AE Blase SL. Rebooting psychotherapy research and practice to reduce the burden of mental illness. Perspect Psychol Sci. (2011) 6(1):21–37. 10.1177/1745691610393527 26162113

[6] Gagnon MP Desmartis M Labrecque M Car J Pagliari C Pluye P Systematic review of factors influencing the adoption of information and communication technologies by healthcare professionals. J Med Syst. (2012) 36:241–77. 10.1007/s10916-010-9473-4 20703721 PMC4011799

[7] Pierce BS Perrin PB McDonald SD. Demographic, organizational, and clinical practice predictors of US psychologists’ use of telepsychology. Prof Psychol Res Pr. (2020) 51(2):184. 10.1037/pro0000267

[8] Aref-Adib G McCloud T Ross J O’Hanlon P Appleton V Rowe S Factors affecting implementation of digital health interventions for people with psychosis or bipolar disorder, and their family and friends: a systematic review. Lancet Psychiatry. (2019) 6(3):257–66. 10.1016/S2215-0366(18)30302-X 30522979

[9] Topol E. The Topol Review. Preparing the healthcare workforce to deliver the digital future. (2019):1–48.

[10] Sammons MT VandenBos GR Martin JN. Psychological practice and the COVID-19 crisis: a rapid response survey. J Health Serv Psychol. (2020) 46(2):51–7. 10.1007/s42843-020-00013-2 32395720 PMC7209971

[11] Sampaio M Navarro Haro MV De Sousa B Vieira Melo W Hoffman HG. Therapists make the switch to telepsychology to safely continue treating their patients during the COVID-19 pandemic. Virtual reality telepsychology may be next. Front Virtual Real. (2021) 36:1–17. 10.3389/frvir.2020.576421 PMC7880047 33585834

[12] Goldschmidt L Langa M Masilela B Ndhlovu LM Mncina B Maubane B Telepsychology and the COVID-19 pandemic: the experiences of psychologists in South Africa. S Afr J Psychol. (2021) 51(2):314–24. 10.1177/0081246321993281

[13] Fairburn CG Patel V. The impact of digital technology on psychological treatments and their dissemination. Behav Res Ther. (2017) 88:19–25. 10.1016/j.brat.2016.08.012 28110672 PMC5214969

[14] Ebert DD Van Daele T Nordgreen T Karekla M Compare A Zarbo C Internet-and mobile-based psychological interventions: applications, efficacy, and potential for improving mental health. Eur Psychol. (2018) 23:167–87. 10.1027/1016-9040/a000318

[15] Sammons MT VandenBos GR Martin JN Elchert DM. Psychological practice at six months of COVID-19: a follow-up to the first national survey of psychologists during the pandemic. J Health Serv Psychol. (2020) 46(4):145–54. 10.1007/s42843-020-00024-z 33225314 PMC7666879

[16] New Zealand Psychologists Board. Core Competencies For the Practice of Psychology in Aotearoa New Zealand. (2018). Available from: https://psychologistsboard.org.nz/wp-content/uploads/2021/06/Core_Competencies.pdf (Accessed May 18, 2022).

[17] Eysenbach G. Improving the quality of web surveys: the checklist for reporting results of internet E-surveys (CHERRIES). J Med Internet Res. (2004) 6(3):e132. 10.2196/jmir.6.3.e34 PMC1550605 15471760

[18] Pote H Moulton-Perkins A. Digital Competencies for Psychological Practitioners in IAPT services. (2020). Available from: https://www.digitalhealthskills.com/digitalcompetencies (Accessed May 18, 2022). 10.2196/92332 PMC13552648 42709962

[19] Braun V Clarke V. Using thematic analysis in psychology. Qual Res Psychol. (2006) 3(2):77–101. 10.1191/1478088706qp063oa

[20] Ministry of Health. Ethnicity data protocols. HISO 10001. Wellington: Ministry of Health (2017).

[21] New Zealand Psychologists Board. Annual Report 1 April 2019–31 March 2020. (2020). Available from: https://psychologistsboard.org.nz/category/annual-report/ (Accessed May 4, 2022).

[22] Wikaire E Harwood M Wikaire-Mackey K Crengle S Brown R Anderson A Reducing healthcare inequities for Māori using Telehealth during COVID-19. N Z Med J. (2022) 135(1552):112–7. https://journal.nzma.org.nz/journal-articles/reducing-healthcare-inequities-for-maori-using-telehealth-during-covid-19-open-access 35728190

[23] Stanford Medicine. Stanford Medicine 2020 Health trends report: The rise of the data-driven physician. Available from: https://med.stanford.edu/content/dam/sm/school/documents/Health-Trends-Report/Stanford%20Medicine%20Health%20Trends%20Report%202020.pdf (Accessed June 28, 2022).

[24] The New Zealand Psychologists Board. The Practice of Telepsychology. (2012). Available from: https://psychologistsboard.org.nz/wp-content/uploads/2021/06/BPG_The_Practice_of_Telepsychology_FINAL_131212.pdf (Accessed May 4, 2022).

[25] Occupational Therapy Board of New Zealand. Competencies for Registration and Continuing Practice for Occupational Therapists. (2022). Available from: https://www.otboard.org.nz/site/ces/competencies?nav=sidebar (Accessed May 4, 2022).

[26] Whittaker R Dobson R Hopley L Armstrong D Corning-Davis B Andrew P. Training clinicians to lead clinical IT projects. N Z Med J. (2020) 133(1572). https://journal.nzma.org.nz/journal-articles/training-clinicians-to-lead-clinical-it-projects 33332334

[27] Honey M Collins E Britnell S. Guidelines: Informatics for nurses entering practice. Auckland: The University of Auckland (2018) 116–22. 10.17608/k6.auckland.7273037.v2

